# Mercury evasion from a boreal peatland shortens the timeline for recovery from legacy pollution

**DOI:** 10.1038/s41598-017-16141-7

**Published:** 2017-11-22

**Authors:** Stefan Osterwalder, Kevin Bishop, Christine Alewell, Johannes Fritsche, Hjalmar Laudon, Staffan Åkerblom, Mats B. Nilsson

**Affiliations:** 1 Department of Forest Ecology and Management, Swedish University of Agricultural Sciences, 901 83 Umeå, Sweden; 2 Department of Environmental Sciences, University of Basel, 4056 Basel, Switzerland; 3 Department of Aquatic Sciences and Assessment, Swedish University of Agricultural Sciences, 75236 Uppsala, Sweden; 4 Department of Earth Sciences, University of Uppsala, 75236 Uppsala, Sweden

## Abstract

Peatlands are a major source of methylmercury that contaminates downstream aquatic food webs. The large store of mercury (Hg) in peatlands could be a source of Hg for over a century even if deposition is dramatically reduced. However, the reliability of Hg mass balances can be questioned due to missing long-term land-atmosphere flux measurements. We used a novel micrometeorological system for continuous measurement of Hg peatland-atmosphere exchange to derive the first annual Hg budget for a peatland. The evasion of Hg (9.4 µg m^−2^ yr^−1^) over the course of a year was seven times greater than stream Hg export, and over two times greater than wet bulk deposition to the boreal peatland. Measurements of dissolved gaseous Hg in the peat pore water also indicate Hg evasion. The net efflux may result from recent declines in atmospheric Hg concentrations that have turned the peatland from a net sink into a source of atmospheric Hg. This net Hg loss suggests that open boreal peatlands and downstream ecosystems can recover more rapidly from past atmospheric Hg deposition than previously assumed. This has important implications for future levels of methylmercury in boreal freshwater fish and the estimation of historical Hg accumulation rates from peat profiles.

## Introduction

Global anthropogenic mercury (Hg) emissions have increased the amount of Hg cycling between vegetation, surface soil, ocean, and the atmosphere by about a factor of three compared to pre-industrial levels^1^. Gaseous elemental mercury (GEM) has an atmospheric lifetime of 0.8–1.7 years after emission, resulting in long range transport before deposition on the earth surface^2^. This poses a threat to humans and wildlife even in remote areas^3^. Current global models assume that Hg deposition to non-contaminated terrestrial surfaces and subsequent re-emission are similar in magnitude. However, large uncertainty remains mostly due to a strong spatial and temporal bias in direct flux measurements towards Hg-enriched sites, and/or short-term, daytime and summertime measurements^4,5^.

Peatlands play an important role in Hg cycling because they constitute a major source of methylmercury to adjacent streams and lakes^6^. Methylmercury is the Hg compound most prone to bioaccumulation in aquatic food webs^3^. The large amount of Hg in peatlands relative to wet bulk deposition and export in surface water runoff indicates that they have accumulated Hg from past atmospheric deposition^7^. In high latitude regions almost all freshwater fish have Hg concentrations exceeding European limits for good ecological status (0.02 mg Hg kg^−1^ fish muscle^8,9^). To predict future Hg levels in fish of these regions, the annual net ecosystem flux of Hg from peatlands needs to be quantified.

Since the store of Hg in peat is so large compared to runoff fluxes, it is generally assumed that it will take several decades, if not centuries, before a reduction in atmospheric deposition could lead to any reduction in the Hg pools of peatlands, thus putting the prospect of substantial recovery far into the future^7,10^. Such calculations, however, neglect GEM emission to the atmosphere which preliminary measurements indicated are substantial^11–13^.

Earlier research has found that below a certain threshold of atmospheric GEM concentration, terrestrial surfaces switch from being net sinks to net sources of Hg^5,14–21^. The exact value of this threshold will vary, though, depending on the nature of the surfaces involved, ambient air characteristics and other physical and chemical conditions^22–24^ or atmospheric turbulence^25,26^. Specific studies have shown that at ambient atmospheric GEM concentrations (~1.5 ng m^−3^), vegetated terrestrial surfaces are net sinks of atmospheric GEM which is incorporated into leaf tissue in forests^18^, grasslands^27^, peatlands^28^, or the arctic tundra^29^. However, Hg land-atmosphere exchange at a peatland scale not only represents uptake of Hg by vegetation but also re-emission of GEM induced by photochemical processes at the peat surface and non-photochemical abiotic reduction by natural organic matter^30–33^. Agnan *et al*.^5^ reported that over barren, litter-covered and vegetated soils, the GEM flux, on average, switches from net deposition to net emission below an atmospheric GEM concentration of 2.75 ng m^−3^.

We hypothesize that recent declines in atmospheric GEM concentrations have fallen below the threshold at which some peatlands switch from being a net sink of atmospheric Hg to being a source of Hg to the atmosphere due to increasing emission of GEM from the peatland surface. If this proves to be the case, the loading of Hg from such peatlands to aquatic food webs would be reduced substantially faster than previously anticipated. To test this hypothesis, we established the first full year, Hg mass balance for a boreal peatland. The key to achieving this was continuous measurement of land-atmosphere GEM exchange. Together with data on export of total mercury (THg) in stream runoff, wet bulk deposition, and peat Hg storage, these measurements allowed us to estimate the major components of the annual Hg mass balance for a boreal peatland.

## Results and Discussion

The most challenging component of a catchment scale Hg mass balance is the long-term continuous measurement of land-atmosphere exchange of GEM. We used a novel, dual-inlet, single detector relaxed eddy accumulation (REA) system to quantify GEM fluxes^13^. REA is a direct measurement approach that overcomes uncertainties associated with other micrometeorological techniques (aerodynamic gradient and modified Bowen-ratio methods) that rely on concentration measurements at different heights. Compared to other GEM-REA systems, the advanced REA design used in this study is fully automated and simultaneously collects GEM emission from, and deposition to, the ground surface^13^.

The system was deployed in the center of an open area of the nutrient poor, minerogenic Degerö Stormyr peatland [64°11′N, 19°33′E] located near Vindeln in the county of Västerbotten, Sweden (see Supplementary Fig. S1). The study area is representative of one of the dominant mire ecosystems in the boreal region. Our study was conducted within the mire complex that is drained by the Vargstugbäcken stream. The catchment covers an area of 2.7 km^2^ of which 70% is peatland^34^. The REA sampling inlets were mounted 3.5 m above the peatland surface, with a ca. 2.5 ha footprint (see Supplementary Fig. S2). Continuous measurements integrating exchange over such a large area overcome much of the measurement uncertainty that would otherwise be introduced with small-scale dynamic flux chambers. Chambers cover only small fractions of the peatland surface and are normally only deployed during short time periods, thus introducing large spatial and temporal uncertainties^5,35^.

**Figure 1:**
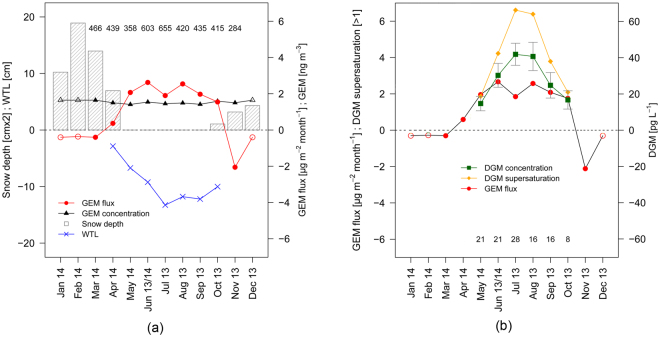
Year-round measurement of gaseous elemental Hg (GEM) concentrations in ambient air, GEM peat-atmosphere flux, water table levels, snow cover, dissolved gaseous Hg (DGM) concentrations in porewater and its supersaturation with respect to atmospheric GEM concentrations at the Degerö Stormyr peatland. **(a)** Monthly average of the net gaseous elemental Hg (GEM) flux, atmospheric GEM concentration, snow depth and water table level (WTL). Negative GEM fluxes represent GEM dry deposition to the peatland, while positive fluxes represent GEM volatilization. Empty symbols represent GEM flux measurements over snowpack measured in March 2014. The figures at the top of the panel represent total numbers of 60-min averages of both GEM flux and atmospheric GEM concentration per month. **(b)** Monthly averages and standard errors of the dissolved gaseous Hg (DGM) concentration measured on a weekly basis between May and October. For comparison, the degree of DGM supersaturation of the peat pore water and the GEM flux is indicated. Number of observations for bulk DGM is displayed.

We found that the annual Hg mass balance at the Degerö Stormyr peatland was clearly dominated by net GEM evasion due to substantial net emission between May and October (Fig. 1a). The annual GEM emission was 9.4 µg m^−2^ and the wet bulk deposition was 3.9 µg m^−2^. GEM is the predominant atmospheric form of Hg and can contribute substantially to net Hg deposition over vegetated surfaces^28,29^. Multi-year gaseous oxidized Hg (GOM) and particulate bound Hg (PBM) concentrations measured at northern European sites in southern Scotland^36^ and the western coast of Sweden^37^ revealed low average levels for GOM < 1 pg m^−3^ and PBM < 4 pg m^−3^. We thus expect that dry deposition of GOM and PBM has only a marginal effect on the Hg mass balance for the Degerö Stormyr peatland. This assumption is confirmed by Enrico *et al*.^28^ who found that dry deposition of GOM and PBM contributed less than 1% to the total annual deposition flux to the Pinet peat bog in the French Pyrenees. This was examined using stable Hg isotope signatures. This clearly indicates that GEM dry deposition and Hg wet deposition are the dominant deposition pathways. These conclusions are valid even when considering the findings by Gustin *et al*.^38^ that demonstrated that GOM concentrations monitored with the instruments used in the studies above (refs^36,37^) are generally underestimated by a factor of 1.6 to 12 depending on the composition of GOM. Even if being ~10 times higher the contribution from GOM will be minor.

**Figure 2:**
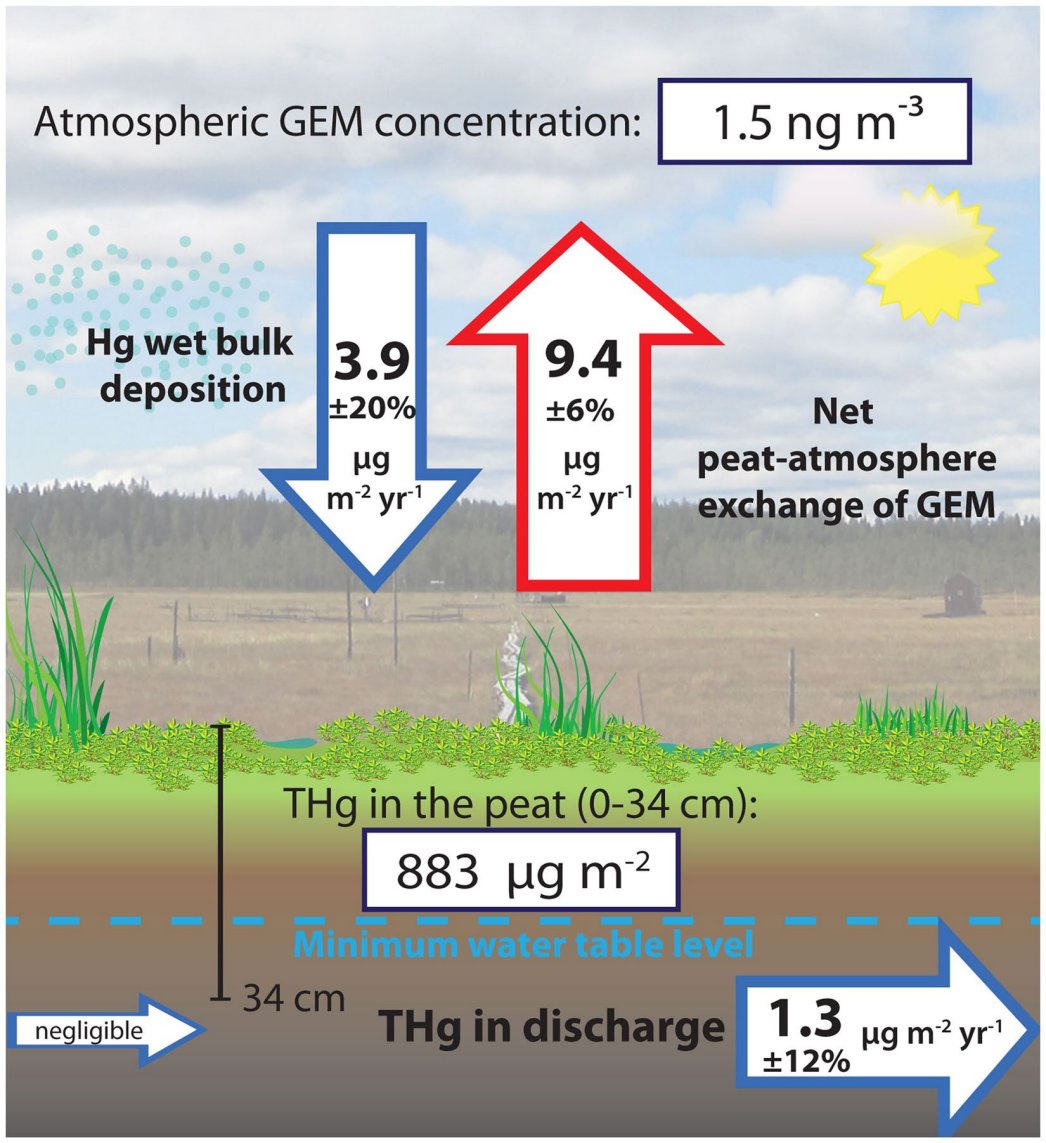
Annual Hg mass balance at the Degerö Stormyr peatland (June 18, 2013 – June 17, 2014). Net emission of gaseous elemental Hg (GEM) and total Hg (THg) in discharge are the major Hg output pathways. Wet bulk deposition is considered as the only Hg input pathway. The units for the flux represented by each arrow are in µg m^−2^ yr^−1^. Uncertainties for GEM flux, total Hg inputs in wet bulk deposition and outputs in stream discharge are indicated (see section “Methods” for details).

Annual discharge export of Hg from the catchment area amounted to 1.3 µg m^−2^ between July 2013 and June 2014 (Fig. 2). The average annual THg export in runoff during the four previous years 2009–2012 was 1.7 µg m^−2^ yr^−1^, ranging between a low of 1.3 µg m^−2^ yr^−1^ in 2010 to a high of 2.3 µg m^−2^ yr^−1^ in 2012. The THg export in discharge has commonly been thought of as the dominant output pathway for most boreal catchments^10,39^. At the study site, however, the peatland-atmosphere evasion was seven times larger than stream discharge of Hg. The stream discharge originates from within the topographically-defined catchment because it is the local high point with no regional groundwater sources contributing to the catchment water balance^34^. This is supported by a previous water balance assessment indicating a low potential for other hydrological sources coming from outside the delineated catchment area^40^. The potential contribution of THg from the mineral soils (30% of total catchment area) to the calculated fluxes is also considered low since dissolved organic carbon (DOC), which is strongly associated with dissolved THg, originates almost entirely from the peatland area of the catchment^41^. This suggestion is supported by: 1) δ^13^C values of DOC in the peat profile near the origin of the outlet stream that are extremely enriched (−5%) compared to measurements in the mineral soils^41^ (−25%) and 2) measurements of the DOC in the peat profile near the stream initiation point for Vargstugbäcken (40.8 ± 12 mg C L^−1^)^41^ compared to the low DOC (~2 mg C L^−1^) in the groundwater from forested podzol soils entering boreal peatlands similar to Degerö Stormyr^42^.

**Figure 3:**
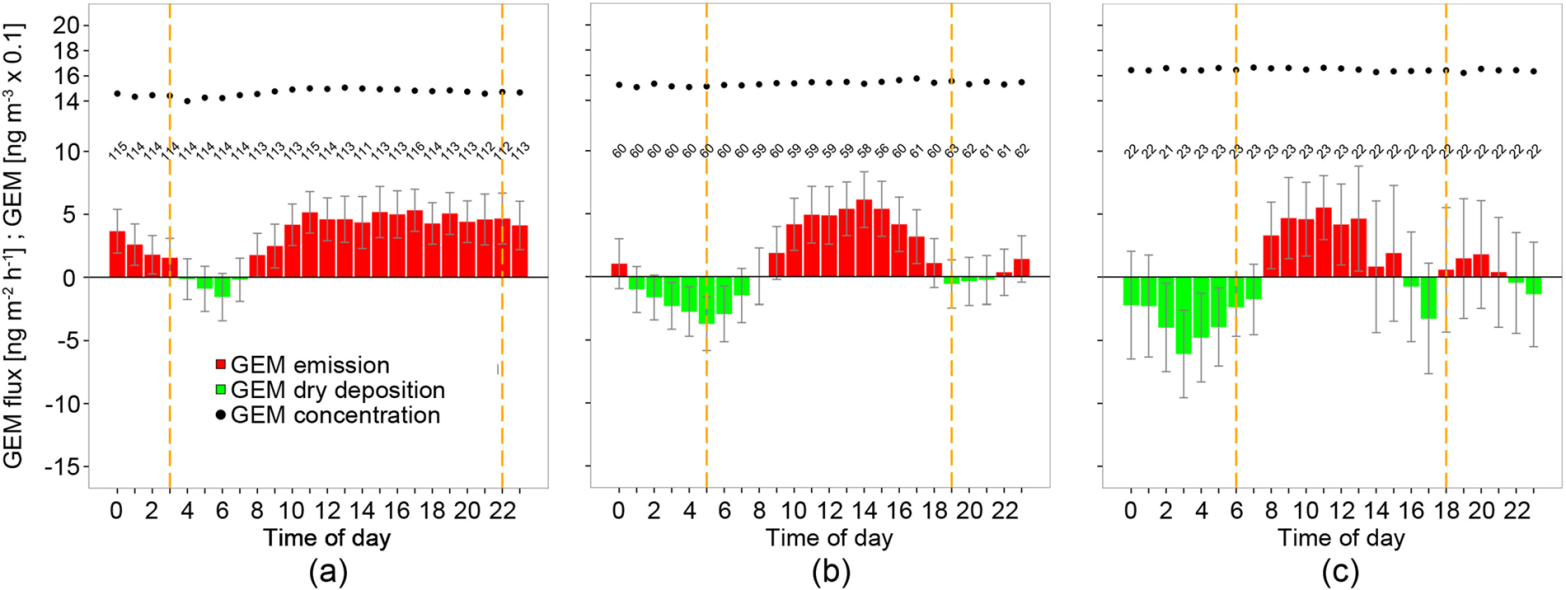
Diel patterns of gaseous elemental Hg (GEM) exchange between the peatland surface and the atmosphere. Flux data were smoothed with a 6-point moving average. The red bars (average ± SE) indicate emission and the green bars dry deposition of gaseous elemental Hg (GEM) during **(a)** the growing season (May-September) **(b)** non-growing season (October, November, and April) and **(c)** snow covered winter time period (represented by data from March). The black dots show the average atmospheric GEM concentrations. Vertical orange dashed lines separate between day (Rg ≥ 5 Wm^−2^) and night (Rg < 5 Wm^−2^). Rg is the hourly average incoming solar radiation. The number of observations of GEM flux and atmospheric GEM concentration are given.

Observed GEM emission during the growing season was 11 µg m^−2^ and substantially exceeded Hg wet bulk deposition during the same time period (2.5 µg m^−2^). During the growing season the peatland emitted GEM to the atmosphere at an average rate ± standard error of the mean (SE) of 3 ± 0.5 ng m^−2^ h^−1^ (Fig. 3a). Outside the growing season (October, November and April) the net GEM flux was close to zero (−0.1 µg m^−2^) with emission dominating from 9 AM to 8 PM and deposition during the night (Fig. 3b). Measurements during March revealed net GEM deposition of 0.4 µg m^−2^ month^−1^ from air to the snow covered surface. Winter time GEM exchange also showed a diurnal pattern indicating photochemical GEM production at the snow surface and subsequent emission of previously deposited GEM in the afternoon (Fig. 3c).

Our data clearly demonstrate the importance of continuous, long-term measurements to derive complete, unbiased annual budgets of the land-atmosphere GEM exchange. There are several factors which corroborate the overall rates of evasion and temporal variation. The largest evasion rates during the summer correspond to peak pore water concentrations of dissolved gaseous mercury (DGM) (Fig. 1b). Field studies over water surfaces have identified photochemically induced Hg^2+^ reduction^43,44^ and dark abiotic reduction^32,45^ as main processes controlling the concentration of DGM in the superficial peat porewater. This suggests the importance of peat porewater DGM concentrations as a driving factor for the net annual peatland-atmosphere exchange of GEM. The near-surface mire porewater was supersaturated in DGM relative to atmospheric GEM concentrations on 86% of the measurement occasions between May and October, peaking in July and August (Fig. 1b).

These findings indicate that the overall mass transfer of GEM from the water-saturated peat layers to the surface is less limited by diffusion than anticipated^23,46,47^. Analysis of GEM concentrations in dry, mineral soils, in contrast, revealed consistent GEM uptake and downward redistribution into the soil^48^ which is indicated by lower soil GEM levels compared to atmospheric GEM concentrations.

Summertime evasion also corresponds to the highest levels of incident radiation that can drive photoreduction of Hg^2+^ to Hg^0^ at the peat surface. Surface chamber measurements over boreal peatlands have demonstrated diel patterns with daytime GEM evasion and nighttime GEM deposition, indicating photochemical reduction at the surface and subsequent GEM evasion^49^. The gross ecosystem production of vascular plants at the measurement site peaks during late June throughout July^50^. The concurrent maximum plant uptake of GEM in July is likely reflected by about 30% lower GEM net emission rates as compared to June and August.

**Figure 4:**
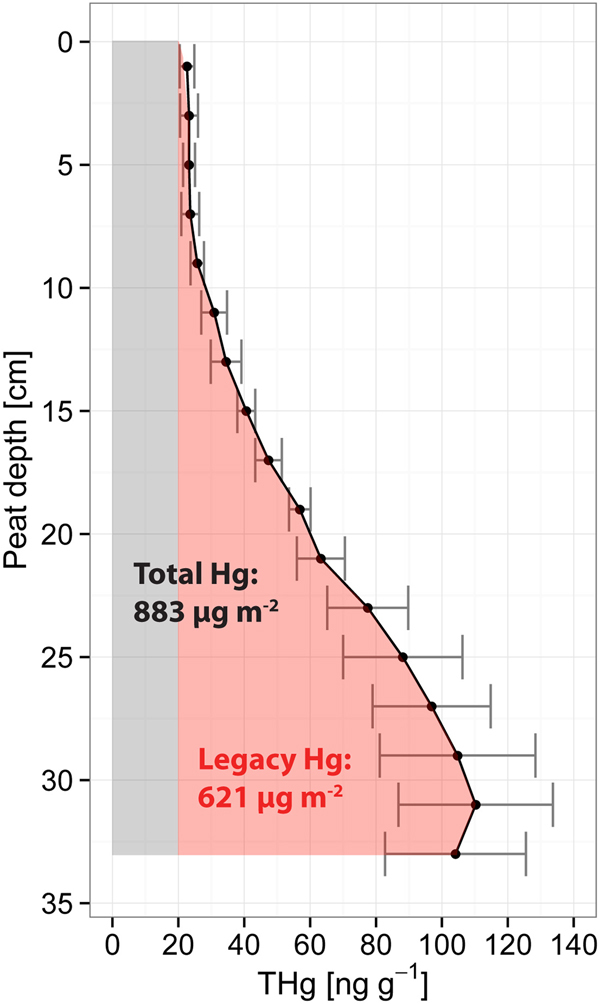
Average depth profile of total Hg (THg) at Degerö Stormyr. THg concentrations from peat cores with 95% confidence interval (n = 8). The zero level denotes the surface of living Sphagnum moss. The red area indicates the amount of legacy Hg stored in the uppermost peat when allowing for a “background” concentration of 20 ng g^−1^.

The unexpectedly high annual net evasion may also be consistent with the pattern of superficial peat Hg concentration-depth profiles. The average ± SE concentration of Hg within the top 34 cm was 57 ± 8 ng g^−1^ dry matter (DM). That is within the range of 10–115 ng g^−1^ DM representing typical soil THg concentrations from boreal forest catchments^51^. Peat THg concentrations increased with depth to a peak of about 110 ng g^−1^ dated to ca 1950 (Fig. 4). This pattern is similar to that of other Hg concentration profiles from open peatlands in Canada^52^, Scotland^53^ and Sweden^54^. This might indicate that the decrease in THg concentration towards the surface (24 ng THg g^−1^ in the top 10 cm) not only represents the degree of peat decomposition and lower Hg deposition rates but also increasing evasion of Hg to the atmosphere after ca 1985. This assumes that the halving of atmospheric GEM concentrations in Europe between ca 1980 and 2000^5,55^ increased evasion of Hg from the peatland surface enough to switch the peatland from being a net sink for Hg to a net source to the surrounding environment, primarily by evasion to the atmosphere.

Since 1990 atmospheric GEM concentration in Western Europe has been decreasing at a rate of 2.2 ± 0.15% yr^−1^ reflecting emission reductions in these regions^56^. The novel Hg flux database presented in Agnan *et al*.^5^ clearly showed that the air concentration of GEM was the only variable that correlated to land-atmosphere exchange at sites with background soil Hg concentrations (r = −0.312, p < 0.001, n = 263). However, even if significant, the negative correlation to air GEM concentrations only explained 11% of the variation in GEM fluxes in this set of data from around the globe. This indicates the importance of local conditions (e.g. different landscape, surface properties and micrometeorology) for the strength and even direction of the GEM flux.

Recent advances in the interpretation of Hg isotope signatures have also revealed high mobility of Hg in peat soils. Jiskra *et al*.^33^ deduced substantial re-emission upon non-photochemical reduction by natural organic matter and estimated that 30% of the historically deposited Hg on their study site has already been re-emitted to the atmosphere. They calculated a peat Hg re-emission flux of 5 µg m^−2^ yr^−1^ that is about 50% of the net GEM flux measured with REA in this study.

The vertical distribution of Hg in surface peat might however also result from decay of organic matter and cannot be interpreted only as a result of changes in Hg deposition and GEM evasion^57^. The surface peat decay rate constant at Degerö Stormyr is 0.0158 yr^−1^ ^58^ resulting in a vertical THg profile similar to the ones observed. However, our results indicate that besides changes in peat mass production and decay processes, GEM evasion has the potential to alter the signal in the vertical peat profile from atmospheric Hg deposition. Potential gaseous losses of Hg and other elements (selenium, sulphur and arsenic) must be carefully considered when interpreting historical and present accumulation rates^59^. As stated by Biester *et al*.^60^ and Jiskra *et al*.^33^, GEM emission provides an alternative explanation for discrepancies between peat and lake sediment archives in the ratio of Hg accumulation rates from preindustrial to modern times^61^.

Accumulation rates of Hg in peat during the industrial period until ca. 1990 are estimated to have been up to 15 times greater than those during the pre-industrial era for northern Europe^62^. Between 1990 and 2011, Hg emissions from European sources decreased by ~60% due to both abatement strategies and the closing of many coal-fired power plants, chlor-alkali plants, etc.^63^. This was associated with roughly a halving of global atmospheric GEM concentrations^55^. In Sweden atmospheric GEM went down from more than 3 ng m^−3^ in the 1980s to northern hemispheric background concentrations of 1.3–1.9 ng m^−3^ after 1995^64,65^.

We hypothesize that this reduction in deposition of atmospheric Hg to terrestrial ecosystems has impacted the bi-directional land-atmosphere exchange of GEM in remote areas. More specifically at Degerö Stormyr, where the current levels of wet bulk Hg deposition are low relative to our observed evasion, the emission of legacy Hg suggests that the peat soil pool is the dominant source for Hg loss from open boreal peatlands. If this situation with high re-emission of GEM is sustained, it will lead to a much faster than anticipated reduction in the pool of Hg from the Degerö Stormyr peatland, and eventually a reduction in the supply of Hg to downstream aquatic food webs.

To make an initial estimate of the time it would take to reduce the supply of Hg to downstream freshwaters, we assumed that the observed levels of evasion will remain constant while no considerable change occurs in either the wet bulk deposition, discharge or the growing season water balance^66^. With a further assumption that catchment runoff is dominated by Hg from the uppermost peat^34,41^, it would take around 90 years to deplete the entire pool of legacy Hg in the uppermost 34 cm to a background concentration level of 20 ng THg g^−1^ (DM) in the peat. Exchange patterns of GEM might vary from year-to-year at the same location and between nearby sites due to changes in meteorological conditions, atmospheric Hg deposition, or plant composition and growth. However, based on our knowledge of carbon cycling, the inter-annual variability of CO_2_ and CH_4_ exchange is limited. The 12-year average ± standard deviation (SD) net ecosystem exchange at Degerö Stormyr was −58 ± 21 g C m^−2^ yr^−1^ ^67^ and the average ± SD (2003–2014) total stream carbon export for a 12-year period was 12.2 ± 3.4 g C m^−2^ yr^−1^ ^34^. Most of the biogeochemical and biogeophysical processes that drive the transformation and transport of the various carbon species are suggested to constitute the major drivers of Hg transformations and transport^39^.We conclude that even with inter-annual and between mire variations, the GEM emission to the atmosphere may constitute a major loss term in the Hg balance of boreal peatlands. This will lead to decreased Hg contamination of downstream aquatic ecosystems in the coming decades, provided that atmospheric Hg levels do not increase. Evasion from peatlands associated with lowered atmospheric GEM levels would thereby attenuate some of the negative effects of past Hg deposition on ecosystems and human health.

The proven reliability of the novel REA system for long-term measurements of land atmosphere GEM exchange^13^ creates possibilities for similar long-term measurements at other peatlands as well as over other ecosystems, including forests and oceans. If the reversal of the net flux of Hg from the atmosphere into peatlands is occurring at a global scale, and possibly even for other ecosystems, then it will increase the societal value of the ambitious goals set by regulatory frameworks such as the UN Minamata Convention for reducing anthropogenic Hg emissions to the atmosphere^68^.

## Methods

### Research site

The annual Hg mass balance was estimated for the period June 18, 2013 – June17, 2014, for the nutrient-poor, boreal mire, Degerö Stormyr (64°11′N, 19°33′E, altitude 270 m above sea level), Västerbotten, Sweden (see Supplementary Fig. S1). The study focused on an open peatland area of 1.9 km^2^ with an average peat depth of 3–4 m (max. 8 m). The GEM flux source area is characterized by uniform microtopography and vegetation, dominated by lawn and carpet plant communities (see Supplementary Fig. S2). The climate is cold and humid with persistent snow cover during ~6 months. The 30-year (1981–2010) mean annual temperature is 1.8 °C and the mean precipitation is 614 mm, of which about 35% falls as snow^69^.

### Peat sampling and Hg analysis

In autumn 2015 two replicate peat profiles were taken in every cardinal direction from the REA tower (N = 8, see Supplementary Fig. S2). The 34 cm long profiles (100 cm^2^ cross section) were cut in 2 cm increments, freeze-dried for 5 days and then weighed for the determination of their dry mass and bulk density (see Supplementary Tab. S1 and Fig. S3). The THg in peat samples was analyzed using a SMS100 (Perkin Elmer, Waltham, USA) through thermal decomposition atomic absorption spectrometry according to EPA method 7473. Certified reference lake sediment (IAEA SL1 [130 ng g^−1^]) and pine needle material (PINE1575a [40 ng g^−1^]) were used for calibration. Replicate samples and the reference material were analyzed regularly (10% of the sampling sequence). The precision was ≤5% relative to the standard deviation.

### Hg in wet bulk deposition

Atmospheric wet bulk deposition was sampled continuously at the EMEP station Bredkälen (63°51′N, 15°20′E; 210 km west of Degerö Stormyr) using IVL wet bulk samplers. Cumulative wet bulk deposition was derived from these THg concentrations and the precipitation at the reference climate station Kulbäcksliden, located 1 km east of Degerö Stormyr (see Supplementary Table S2).

### Peatland-atmosphere exchange of gaseous elemental Hg

The dual-inlet, single detector REA system was set up in the center of the peatland (15 m south of a fully equipped EC tower^70^). Sampling inlets were mounted 3.5 m above the ground. For a more detailed description see Supplementary Fig. S4 and in Osterwalder *et al*.^13^). The REA sampling interval was 30 min and the data coverage during the nine months of measurements was 33%. Between December 2013 and February 2014 no measurements were performed. Monthly averages of GEM flux were computed based on an annual total of 4,075 60-min values. The numbers of hourly GEM flux observations were well balanced over 24 hours (see Fig. 3). To determine the net annual GEM flux we calculated averages for each of the 24 hours during the day for each month. This yielded the diurnal pattern for that month. The sum of this diurnal curve is then multiplied by the number of days for the integrating time period. The annual sum of GEM fluxes is then derived by cumulated monthly averages. The GEM flux source area covered 2.5 ha of homogenous wet lawns and carpet plant communities during snow-free conditions. The Hg detector and the two gold-cartridge pairs were calibrated every second month by injection of different volumes of Hg saturated air from a temperature controlled Hg vapor calibration unit (Tekran Model 2505, Toronto, Canada). GEM recovery was monitored automatically using the GEM reference gas and Hg zero-air generator. The system was regularly set into a reference mode (2s-simulated wind signal) to check for sampling line bias and to investigate the precision of concentration difference measurements. The detection limits were derived from the absolute standard deviation of the residuals from orthogonal linear regression fitting^71^. Residuals (N = 960) did not show any trend with time or GEM concentration. The detection limit (1σ) was 0.05 and 0.04 ng m^−3^ for gold cartridge Pair 1 and Pair 2, respectively. In total 53% and 52% of the measured GEM differences were above these limits. Please note that the average flux values reported in the main text include the measured data below the detection limit (average exchange rates would otherwise be overestimated).

### Hg in catchment discharge

Continuous discharge was measured in a heated flume at the catchment outlet averaged to hourly values. Stream water was sampled twelve times (monthly) for THg during the measurement period. The THg analyses were performed at the Stockholm University Department of Applied Environmental Science following the US EPA standard method 1631 (US EPA, 2002) using a 10.035 Millennium Merlin 1631 CV-AFS (PSA, Orpington, UK). Annual THg export in discharge was calculated by multiplying the interpolated daily THg concentration with daily discharge quantity (see Supplementary Table S3 and Fig. S5).

### Dissolved gaseous Hg and other environmental parameters

Dissolved gaseous elemental Hg concentration in the pore water was measured weekly during June – October 2013 and May–June 2014 using a Tekran Automated Purging System developed and described in Lindberg *et al*.^72^. Instruments to determine meteorological parameters were mounted at 2 m height above the mire surface on the same tower as the eddy covariance system. Air temperature and humidity were determined by an MP100 temperature and moisture sensor (Rotronic AG, Bassersdorf, Switzerland) equipped with a ventilated shield. Global radiation was measured using a Li200sz sensor (LI-COR, Lincoln, USA). Water table levels were measured in a lawn plant community using a float and counterweight system attached to a potentiometer^73^. The snow depth was measured by a Sr-50 ultrasonic sensor (Campbell Scientific Logan, USA) mounted on the REA tower.

### Uncertainty estimates for the peatland Hg mass balance

The uncertainty of the annual land-atmosphere GEM flux was calculated by error propagation of the hourly averages for each day of every month. The monthly errors were then propagated to get an estimated uncertainty of the annual GEM flux which was ±6%. The uncertainty associated with the precipitation inputs was ±20%. This is a conservative estimate since annual deposition values are composed of 26 biweekly measurements where the uncertainty of each Hg analysis is ±7% for concentrations >0.25 ng/L according to the analytical laboratory performing these analyses (IVL Swedish Environmental Research Institute). The uncertainty of the Hg output via catchment discharge was ±12%. That value was estimated using a propagation of error calculation that includes the error in discharge measurements and the error in the THg concentration measurements in runoff (±7%). The nine year inter-annual variation in discharge and total carbon export (sum of DOC, DIC, CO_2_ and CH_4_) are very similar. For discharge the standard errors were ±10% and for total C export ±9%, using the same method applied in this paper to estimate the uncertainty in the export of Hg in discharge^34^. The uncertainty of the peatland Hg budget is ±24%.

## Electronic supplementary material

Supplementary Information
