# Supplementary material for: Mercury evasion from a boreal peatland shortens the timeline for recovery from legacy pollution
Source: Sci Rep. 2017 Nov 22;7:16022. doi: 10.1038/s41598-017-16141-7 (PMC5700116; doi:10.1038/s41598-017-16141-7)
Supplement: Supplementary file 1 — Supplementary Information [file 41598_2017_16141_MOESM1_ESM.pdf]

Supplementary Information

Mercury evasion from a boreal peatland shortens the timeline for recovery from legacy pollution

Stefan Osterwalder[1,2*], Kevin Bishop[3,4], Christine Alewell[2], Johannes Fritsche[2], Hjalmar Laudon[1], Staffan Åkerblom[3], and Mats B. Nilsson[1]

[1] Department of Forest Ecology and Management, Swedish University of Agricultural Sciences, 901 83 Umeå, Sweden
[2] Department of Environmental Sciences, University of Basel, 4056 Basel, Switzerland
[3] Department of Aquatic Sciences and Assessment, Swedish University of Agricultural Sciences, 75236 Uppsala, Sweden
[4] Department of Earth Sciences, University of Uppsala, 75236 Uppsala, Sweden
**Correspondence to: Stefan Osterwalder*

Supplementary Notes

Research site and REA footprint

The Degerö catchment is predominantly drained by the stream Vargstugbäcken northwest of the REA tower (Fig. S1). The soils on the 1.9 km^2 catchment are 70% peat and 30% mineral soil (glacial till). The potential contribution of THg from the mineral soils to the calculated fluxes is considered low due to the spatial arrangement of the till and peat relative to the stream. The REA system was set up in the eastern part of the catchment (Fig. S1). The micro-topography within the source area of the gaseous elemental mercury (GEM) flux is characterized mainly by carpets and lawns, with sparse occurrences of hummocks. The vascular plant community in this area was dominated by *Eriophorum vaginatum* L., *Trichophorum cespitosum* L. Hartm., *Vaccinium oxycoccos* L., *Andromeda polifolia* L., and *Rubus chamaemorus* L. On the bottom of the carpets *Sphagnum majus* C. Jens. is prevalent while *Sphagnum balticum* C. Jens. dominates the lawn. On the hummocks *Sphagnum fuscum* Schimp. Klinggr. and *Sphagnum rubellum* Wils. are the most common mosses[74,75]. In order to confirm the homogeneity of the footprint, i.e. to assure that forest areas do not considerably influence the GEM source area, the REA footprint (Fig. S2b) was calculated using a Lagrangian stochastic forward model[76]. Data were based on measurements between March 7 and June 26, 2014. 50%, 80% and 95% of the total GEM flux source area covered 0.4, 2.5 and 10.1 ha, respectively. The roughness length was set to 0.02 m. The main wind direction during periods when the footprint was calculated was SW (Fig. S2a).

Hg in the peat

The pool of total Hg (THg) in the peat was calculated from eight depth profiles of measured THg concentrations and bulk density (Fig. S3 and Tab. S1). The peat dating was derived from mean vertical peat growth rate at Degerö determined by Olid *et al.*[58]. For the period from 2003 to 2015 a peat growth rate of 0.8 cm yr^{-1} was applied. In the older peat layers (> 12 yr) the vertical growth rate values were 0.4 cm yr^{-1}.

Hg in wet bulk deposition

Cumulative atmospheric wet bulk deposition of Hg for Degerö was derived from wet bulk deposition data from Bredkälen (3.9 µg m^{-2} yr^{-1}), located 210 km west of Degerö and from precipitation data at Degerö Stormyr (Supplementary Table S2). Hg wet bulk deposition was within the range of

measurements conducted at Råö (5.6 µg m^{-2} yr^{-1}) and Pallas (1.5 µg m^{-2} yr^{-1}), located ~850 km southwest and ~470 km northeast of Degerö, respectively. Continuous Hg wet bulk measurements at Råö (57°24'N, 11°55'E), Bredkälen (63°51'N, 15°20'E) and Pallas (67°58'N, 24°07'E) were conducted by the Swedish Environmental Research Institute (IVL) as part of the European Monitoring and Evaluation Programme (EMEP).

Peat-atmosphere flux of gaseous elemental Hg

Vertical wind velocity (w) was measured with a USA-1 sonic anemometer (Metek, Elmshorn, Germany) at 3.5 m height. Its signal determined the switching frequency of the fast-response solenoid valves that isolated GEM in up- and downdrafts. GEM was collected on two pairs of gold cartridges and analyzed every 30 min with a CVAFS Hg detector (Tekran Model 2500, Toronto, Canada). The turbulent GEM flux (ng m^{-2} h^{-1}) was computed by multiplying the standard deviation of the vertical wind velocity with the difference between the average GEM concentration in updrafts and downdrafts and the relaxation coefficient β. β was derived from concurrent eddy covariance measurements of sensible heat flux for each averaging period. Bias in vertical wind measurements was removed through the application of a recursive high-pass filter. The air was drawn through 8 m long Teflon® PTFE sampling tubes and was sampled at a flow rate of 1.5 L min^{-1}. The system was placed within a dome shaped plexiglass container (Igloo Satellite Cabin, Icewall One, Australia), which also houses the main part of ICOS instrumentation.

The system hardware was improved as suggested in a previous study[13]: (i) the Hg detector and the Hg reference gas generator were kept at a constant ambient temperature of 25°C, (ii) a pair of pressure sensors and a second set of fast response valves (V1.2, V2.2, V2.3) were implemented to better control pressure fluctuations and (iii) the up-and downdraft air volumes were now measured with two high precision thermal mass flow meters (Vögtlin Instruments, Aesch, Switzerland) (Fig. S4).

The analyzed air samples were corrected for changes in sensitivity of the gold cartridges and the Hg detector. The GEM concentrations were calculated considering results from the manual calibration procedure and air volumes drawn over the gold cartridges (minimum of 3 liters per gold cartridge). They were discarded when differing more than three times the standard deviation of their moving average (window width of 48 observations). GEM concentrations were corrected for the bias between

the two sampling lines by interpolation between reference mode sampling. GEM fluxes were discarded due to poorly developed turbulence[13,77] and in general during periods of very stable atmospheric conditions (z/L > 2). Short gaps in the flux time series smaller than 9 observations were filled using a standard look-up table (LUT) method[78]. Therefore, a LUT with 6 fixed intervals for solar radiation was used to “look up” GEM flux at similar meteorological conditions. The total number of 30-min observations during growing- and non-growing season and the snow season was 3,129, 1,560 and 466 respectively.

Hg in catchment discharge

Stream discharge at the catchment outlet (Fig. S1) has been measured continuously at a flume within a heated hut since 2008. For this study THg in catchment discharge was measured between June 18, 2013 and June 17, 2014 (Fig. S5). Stream water was sampled in high-density polyethylene bottles on a monthly basis. The THg samples were preserved, within 24 - 48 h after the sampling, by adding 0.7 mL suprapur HNO_3 to the 125 mL samples.

THg analysis was performed by the Stockholm University Department of Applied Environmental Science on unfiltered water samples by using cold vapor atomic fluorescence spectroscopy (CV-AFS) after oxidation by BrCl and reduction to Hg^0 with $SnCl_2$. The detection limit was 0.3 ng L^{-1} and the analytical precision was ±3% for THg in a concentration range of 5 - 50 ng L^{-1}.

The THg concentrations did not correlate with the total organic carbon content in the discharge on the day of sampling (data not shown). To calculate the total loss of THg from the Degerö catchment in stream discharge (Tab. S3), stream THg concentrations were interpolated linearly between sampling occasions and multiplied by the daily discharge.

Atmospheric wet bulk deposition and GEM uptake by plants are the only new Hg sources to the peatland catchment that can offset either emission to the atmosphere or discharge from the catchment. The diffuse flow of water from mineral soils surrounding the peatland into the peatland itself mostly occurs far from the stream outlet. Due to the strong binding properties of peat for metals, the metals in groundwater from the mineral soil areas are generally held in the organic peat directly adjacent to where the water from the mineral soil enters the peat in the “lagg” zone[42].

Dissolved gaseous Hg in the peat pore water

Once a week during June - October 2013 and May - July 2014 four peat pore water samples were taken from the same areas as the peat cores were extracted (Fig. S2b). Pore water was sucked from the water table level down to 10 cm using a Teflon® FEP pole connected to a rotary vane pump and collected in a 1 L Teflon® PFA vessel protected from sunlight to eliminate photolytic reactions. Before sampling the vessel was acid washed, cleaned with ultrapure water (Milli-Q, 18.2 MΩ·cm, Millipore Corp.) and rinsed with peat pore water. Polyethylene gloves were worn during the sampling and analysis. The vessels were immediately transferred to the onsite field laboratory (< 5 min) and placed in a water bath protected from sunlight with temperatures kept close to the temperature of the peat pore water. The samples were then analyzed using a Tekran Automated Purging System according to Lindberg *et al.*[72].

For purging, the inlet line to the Hg analyzer (Tekran Model 2537A, Toronto, Canada) was connected to the vessel outlet at the beginning of a measurement sequence. The outlet airstream was mixed with a stream of Hg-free air (0.2 L min^{-1}). The Hg analyzer was set to a flow rate of 1.2 L min^{-1} and purged the vessel at a rate of 1 L min^{-1}. Twelve, 5 min purge samples were collected for a total of 60 min (40 min sample, 20 min whole-blank). At the vessel inlet an activated carbon trap supplied Hg-free air. The flow rates were continuously controlled using digital mass flow controllers (Red-y compact series, Vögtlin, Aesch, Switzerland) to identify potential leaks during the measurement. The blanks were tested prior to every sampling by purging the vessel filled with ultrapure water. The measurements declined immediately to values below the detection limit (~0.5 pg).

The DGM concentration was calculated from the difference between the sample that was purged free of DGM and the whole-blank purge. This difference was then expressed on a water volume basis. The average whole-blank signal was 2.5% and was always below 9%. The DGM concentrations showed a distinct seasonal pattern and ranged from 2 pg L^{-1} (May 2014) to 82 pg L^{-1} (July 2014). In 86% of the measurements, the peatland pore water was supersaturated, relative to the atmosphere, with DGM. The degree of saturation is calculated by the ratio of Henry's law constant times DGM concentration divided by the atmospheric GEM concentration (Eqs. in Gårfeldt *et al.*[79]).

Data analysis and visualization

Statistical analysis was performed with R, version 3.4.0 (R Foundation for Statistical Computing, Vienna, Austria) using RStudio 1.0.153 (RStudio Inc., Boston, USA). Figure 2 in the main text was created using Adobe Illustrator CS5 (Version 15, Adobe, San Jose, USA).

Supplementary Figures

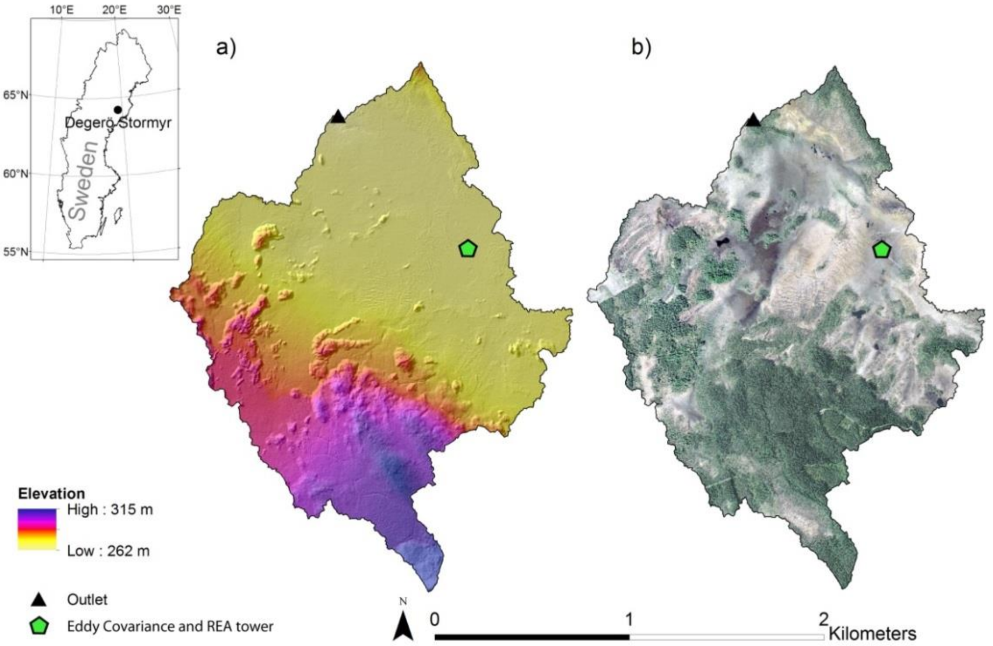

Figure S1. Map of the Degerö Stormyr catchment, located in northern Sweden (from Leach *et al.*[34]; we acknowledge the permission of the publisher to reproduce this figure here). Digital elevation model of the catchment derived from LiDAR **(a)** and aerial photo **(b)**. The location of the eddy covariance and REA tower is indicated by a green pentagon. The black triangle indicates the catchment outlet. GIS data sources are from the Swedish Mapping, Cadastral and Land Registration Authority (Lidar data, Laserdata © Lantmäteriet (2010); Aerial photograph, GSD-Ortofoto © Lantmäteriet (2004); Overview map, GSD-Översiktskartan © Lantmäteriet (2004)).

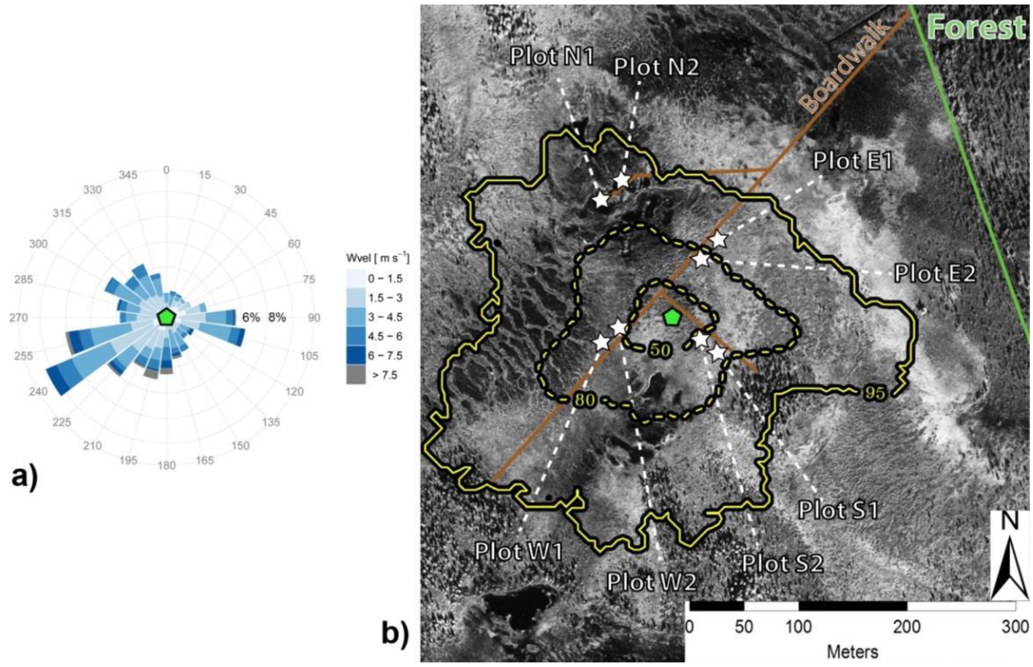

Figure S2. Wind rose and aerial photograph of Degerö Stormyr with contours bordering the Hg source area. Polar histogram of the 30 min averaged wind speed (m s^{-1}) measured at 3.5 m above the surface between March 07 and June 26, 2014 **(a)**. Aerial photograph with yellow contours containing 50%, 80% and 95% of the REA footprint calculated using 2,841 half hourly flux values for the same period of time. Eight plots are indicated where the peat cores were taken and dissolved gaseous Hg (DGM) was analyzed. The green pentagon indicates the location of the REA tower **(b)**. The aerial photograph (GSD-Ortofoto © Lantmäteriet (2004)) was processed using Adobe Photoshop CS5 (Version 12.0, Adobe, San Jose, USA).

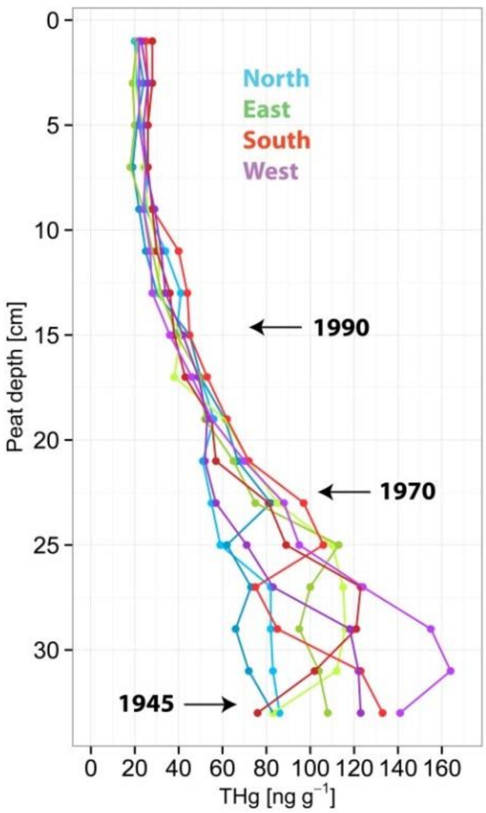

Figure S3. Depth profile of total Hg (THg) concentrations from eight peat cores. Two cores were taken in all four cardinal directions. The peat age was dated after Olid *et al.*[58]

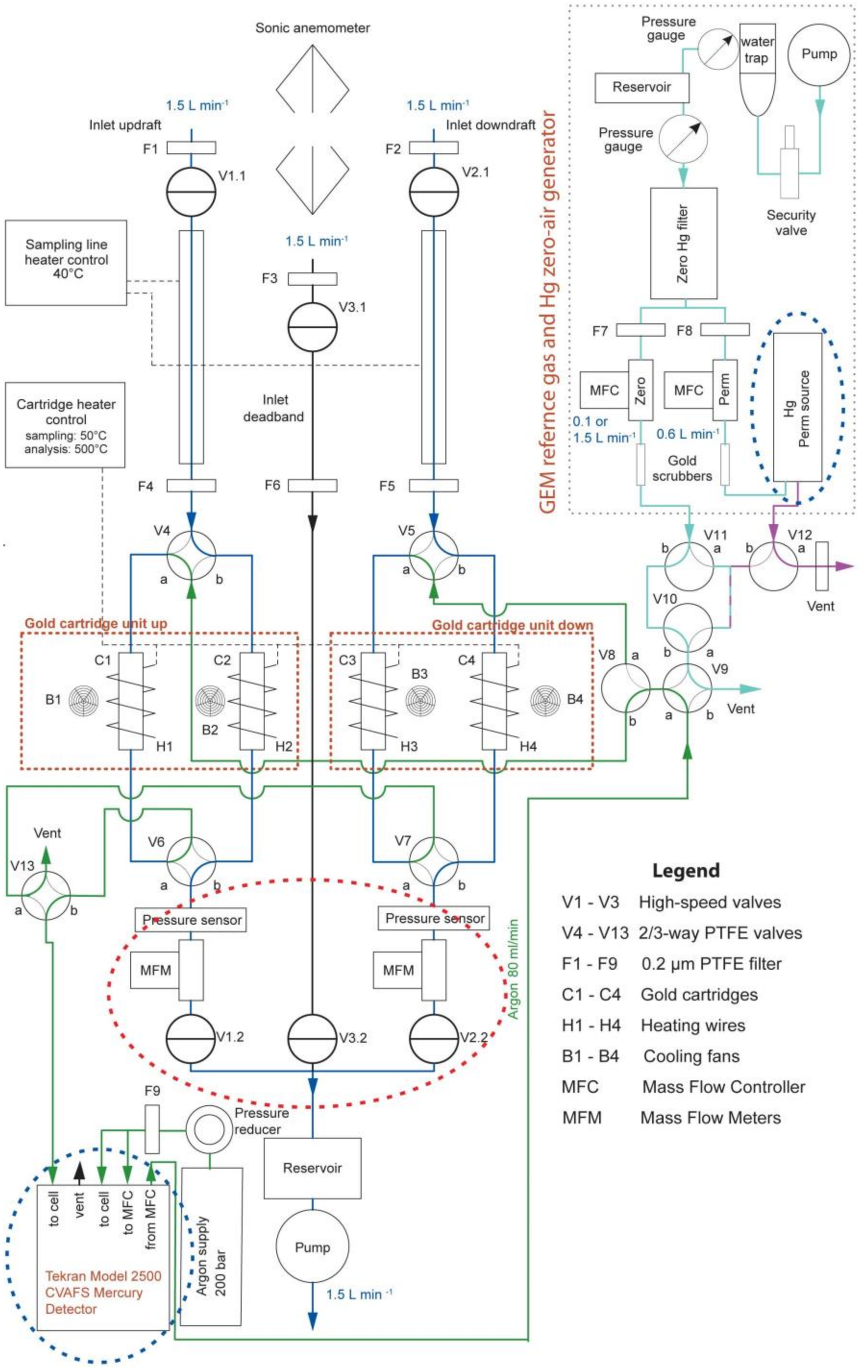

Figure S4. Schematic of the REA system hardware. The system consisting of a gaseous elemental Hg (GEM) sampling unit, a GEM reference gas and Hg zero-air generator (upper right). The major novelties compared to the system presented in Osterwalder *et al.*[13] are marked with dashed ellipses.

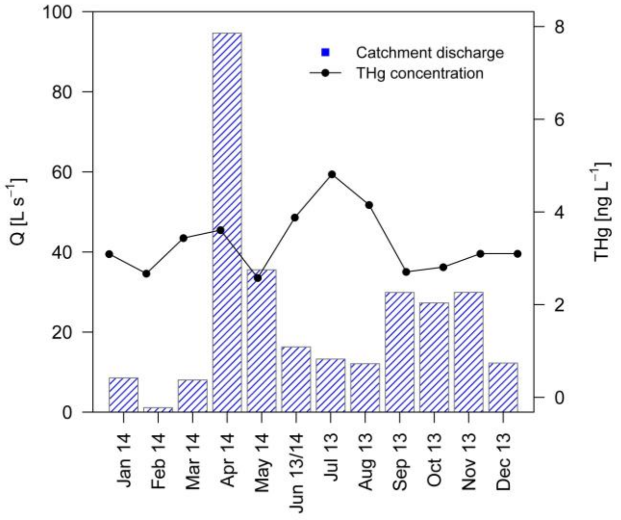

Figure S5. Mercury in catchment discharge. Monthly averages of catchment discharge (Q) and total Hg (THg) concentration in the discharge.

Supplementary Tables

Table S1. Mercury depth profile derived from eight peat cores. Mean ± SE (n=8) concentration profile of THg (THg_C) and stored Hg within each depth interval (THg_S). Linear depth, bulk density, estimated peat age and accumulation rate of Hg (HgAR) are also given. [a] dated after ref (58)

Depth (cm)	THg_C (ng g^{-1})	THg_S (µg m^{-2})	Bulk density (g cm^{-3})	year[a]	HgAR (µg m^{-2} yr^{-1})
0-2	22.6 ± 0.9	10.0 ± 0.7	0.022	2014	4
2-4	23.3 ± 1.1	8.7 ± 0.9	0.019	2011	3
4-6	23.3 ± 0.8	9.7 ± 0.8	0.021	2009	4
6-8	23.6 ± 1.1	10.4 ± 0.7	0.022	2006	4
8-10	25.8 ± 0.9	13.0 ± 0.9	0.026	2004	5
10-12	30.9 ± 1.7	17.8 ± 1.2	0.029	1999	4
12-14	34.5 ± 2.0	23.6 ± 2.5	0.034	1994	5
14-16	40.6 ± 1.1	30.3 ± 3.3	0.038	1989	6
16-18	47.4 ± 1.7	34.8 ± 3.1	0.037	1984	7
18-20	56.9 ± 1.4	47.9 ± 4.6	0.042	1979	10
20-22	63.3 ± 3.1	57.3 ± 5.8	0.045	1974	11
22-24	77.5 ± 5.2	77.1 ± 9.4	0.048	1969	15
22-26	88.1 ± 7.7	91.4 ± 11.9	0.051	1964	18
26-28	96.9 ± 7.6	99.7 ± 13.3	0.051	1959	20
28-30	104.8 ± 10.0	100.7 ± 12.2	0.049	1954	20
30-32	110.3 ± 9.9	122.6 ± 15.8	0.055	1949	25
32-34	104.1 ± 9.0	127.5 ± 12.3	0.063	1944	26

Table S2. Wet bulk deposition of mercury. Precipitation (Precip), total mercury concentrations in the precipitation samples (Hg_P) and total mercury in wet bulk deposition (Hg_D) measured at Bredkälen. Wet bulk deposition of Hg for the Degerö Stormyr catchment (Hg_D) was derived from precipitation measurements 1 km east of Degerö Stormyr.

**Timespan**		**Bredkälen**			**Degerö**	
		Precip (mm)	Hg_P (ng L^{-1})	Hg_D (ng m^{-2})	Precip (mm)	Hg_D (ng m^{-2})
06/18/2013	07/02/2013	80.1	5.9	252.2	60.3	354.4
07/02/2013	07/30/2013	96.9	12.7	1234.9	51.5	656.3
07/30/2013	09/03/2013	88.0	5.5	484.9	91.9	506.4
09/03/2013	10/01/2013	97.2	2.4	229.1	69.1	162.9
10/01/2013	10/29/2013	32.6	2.8	92.1	47.7	134.7
10/29/2013	12/03/2013	24.5	3.1	76.7	47.4	148.5
12/03/2013	12/31/2013	26.0	4.0	103.3	64.9	257.9
12/31/2013	02/04/2014	9.7	11.4	110.6	36.4	415.0
02/04/2014	03/04/2014	24.2	6.7	163.1	62.1	418.5
03/04/2014	04/01/2014	24.9	6.1	152.4	13.9	85.1
04/01/2014	04/29/2014	1.8	42.4	76.3	1.8	76.3
04/29/2014	06/03/2014	80.0	7.1	564.3	50.9	359.0
06/03/2014	06/17/2014	55.2	12.4	332.6	23.1	324.8

Table S3. Mercury in catchment discharge. Monthly total of mercury in discharge (THg) and total discharge from the catchment.

Month	THg ($ng\ m^{-2}$)	Discharge (10^6 L)
Jul 13	89.9	35.5
Aug 13	70.6	32.4
Sep 13	107.2	77.5
Oct 13	111.2	73
Nov 13	126.6	77.6
Dec 13	53.3	32.8
Jan 14	37.3	22.9
Feb 14	3.8	2.7
Mar 14	39.9	21.6
Apr 14	463.3	245.3
May 14	129.5	95.2
June 13/14	73.6	41.4
Annual total	1,307	759

References

13. Osterwalder, S. *et al.* A dual-inlet, single detector relaxed eddy accumulation system for long-term measurement of mercury flux. *Atmos. Meas. Tech.* **9**, 509-524 (2016).
34. Leach, J. A., Larsson, A., Wallin, M. B., Nilsson, M. B. & Laudon, H. Twelve year interannual and seasonal variability of stream carbon export from a boreal peatland catchment. *J. Geophys. Res. Biogeosci.* **121**, 2016JG003357 (2016).
42. Lidman, F., Köhler, S. J., Mörth, C.-M. & Laudon, H. Metal transport in the boreal landscap - the role of wetlands and the affinity for organic matter. *Environ. Sci. Technol.* **48**, 3783-3790 (2014).
58. Olid, C., Nilsson, M. B., Eriksson, T. & Klaminder, J. The effects of temperature and nitrogen and sulfur additions on carbon accumulation in a nutrient-poor boreal mire: Decadal effects assessed using 210Pb peat chronologies. *J. Geophys. Res.-Biogeosci.* **119**, 392-402 (2014).
72. Lindberg, S. E., Vette, A. F., Miles, C. & Schaedlich, F. Mercury speciation in natural waters: Measurement of dissolved gaseous mercury with a field analyzer. *Biogeochemistry* **48**, 237-259 (2000).
74. Nilsson, M. *et al.* Contemporary carbon accumulation in a boreal oligotrophic minerogenic mire - a significant sink after accounting for all C-fluxes. *Glob. Change Biol.*, **14**, 2317-2332 (2008).
75. Laine, A. M. *et al.* Abundance and composition of plant biomass as potential controls for mire net ecosystem CO_2 exchange. *Botany*, **90**, 63-74 (2012).
76. Rannik, U. *et al.* Footprint analysis for measurements over a heterogeneous forest, *Bound.-Lay. Meteorol.* **97**, 137-166 (2000).
77. Foken, T. *Angewandte Meteorologie, Mikrometeorologische Methoden*, Berlin, 2. Auflage, Springer, Berlin (2006).
78. Falge, E. *et al.* Gap filling strategies for defensible annual sums of net ecosystem exchange. *Agricultural and Forest Meteorology* **107**, 43-69 (2001).
79. Gårdfeldt, K., Feng, X. B., Sommar, J. & Lindqvist, O. Total gaseous mercury exchange between air and water at river and sea surfaces in Swedish coastal regions. *Atmos. Environ.* **35**, 3027-3038 (2001).
